# Airborne Mold and Endotoxin Concentrations in New Orleans, Louisiana, after Flooding, October through November 2005

**DOI:** 10.1289/ehp.9198

**Published:** 2006-06-12

**Authors:** Gina M. Solomon, Mervi Hjelmroos-Koski, Miriam Rotkin-Ellman, S. Katharine Hammond

**Affiliations:** 1 Natural Resources Defense Council, San Francisco, California, USA; 2 Division of Occupational and Environmental Medicine, Department of Medicine, University of California, San Francisco, California, USA; 3 Institute of Arctic and Alpine Research, University of Colorado, Boulder, Colorado, USA; 4 School of Public Health, University of California, Berkeley, California, USA

**Keywords:** air quality, bioaerosols, endotoxin, flood, Katrina, mold, New Orleans

## Abstract

**Background:**

The hurricanes and flooding in New Orleans, Louisiana, in October and November 2005 resulted in damp conditions favorable to the dispersion of bioaerosols such as mold spores and endotoxin.

**Objective:**

Our objective in this study was to assess potential human exposure to bioaerosols in New Orleans after the flooding of the city.

**Methods:**

A team of investigators performed continuous airborne sampling for mold spores and endotoxin outdoors in flooded and nonflooded areas, and inside homes that had undergone various levels of remediation, for periods of 5–24 hr during the 2 months after the flooding.

**Results:**

The estimated 24-hr mold concentrations ranged from 21,000 to 102,000 spores/m^3^ in outdoor air and from 11,000 to 645,000 spores/m^3^ in indoor air. The mean outdoor spore concentration in flooded areas was roughly double the concentration in nonflooded areas (66,167 vs. 33,179 spores/m^3^; *p* < 0.05). The highest concentrations were inside homes. The most common mold species were from the genera of *Cladosporium* and *Aspergillus*/*Penicillium; Stachybotrys* was detected in some indoor samples. The airborne endotoxin concentrations ranged from 0.6 to 8.3 EU (endo-toxin units)/m^3^ but did not vary with flooded status or between indoor and outdoor environments.

**Conclusions:**

The high concentration of mold measured indoors and outdoors in the New Orleans area is likely to be a significant respiratory hazard that should be monitored over time. Workers and returning residents should use appropriate personal protective equipment and exposure mitigation techniques to prevent respiratory morbidity and long-term health effects.

Hurricane Katrina struck New Orleans, Louisiana, on 29 August 2005, breaching the levees that protected the city and flooding approximately 120,000 homes. Some homes remained underwater for weeks, and some neighborhoods were flooded again when Hurricane Rita struck on 22 September 2005. Flood damage varied throughout the city, with some areas spared and others flooded to the rooflines. There were immediate concerns about environmental health hazards ranging from oil spills and disruptions of hazardous waste sites, to sewage contamination in flood-waters (U.S. Environmental Protection Agency 2006). As the water receded, concerns related to air quality emerged—specifically, aerosolization of mold spores and endotoxin. A survey by the Centers for Disease Control and Prevention (CDC) in late October found that 46% of randomly selected homes in the New Orleans area had visible mold growth, and 17% had heavy mold coverage (CDC 2006).

## Mold

Filamentous microfungi (mold) can threaten human health through release of spores that become airborne and can be inhaled. Some molds produce metabolites (mycotoxins) that can initiate a toxic response in humans or other vertebrates (Robbins et al. 2000). Repeated exposure to significant quantities of fungal material can result in respiratory irritation or allergic sensitization in some individuals (Bush et al. 2006). Sensitized individuals may subsequently respond to much lower concentrations of airborne fungal materials. Of the thousands of types of fungal spores found in indoor and outdoor environments, adverse health effects in humans have most frequently been associated with *Alternaria*, *Aspergillus, Cladosporium, Penicillium*, and *Stachybotrys* (Hossain et al. 2004; Jarvis and Miller 2005; O’Driscoll et al. 2005; Stark et al. 2003).

In outdoor air, elevated concentrations of fungal spores are associated with allergic and asthmatic responses in humans. A large Canadian time-series study reported that daily fluctuations in ambient mold spores are directly associated with childhood asthma attacks requiring a visit to an emergency department (Dales et al. 2004). Researchers in Southern California reported an association between ambient mold spore concentrations and childhood asthma attacks even in areas where the airborne spore concentrations are relatively low (12-hr daytime mean spore concentration of ~ 4,000 spores/m^3^) (Delfino et al. 1997).

The health effects of exposure to mold in the indoor environment have been extensively studied (Belanger et al. 2003; Portnoy et al. 2005). An Institute of Medicine (IOM) committee concluded in 2004 that there is sufficient evidence of a causal link between indoor dampness and upper respiratory tract symptoms, cough, wheeze, asthma symptoms in sensitized people, and hypersensitivity pneumonitis in susceptible people (IOM 2004). The panel further concluded that there is suggestive evidence of an association between damp indoor environments and dyspnea, lower respiratory illness in healthy children, and new-onset asthma. Although the IOM report did not ascribe all of these health effects to mold, the committee noted that dampness and mold are highly intercorrelated. A more recent large population-based prospective cohort study found that the presence of mold odor in the home was associated with a 2.4-fold increased incidence rate of asthma among children (Jaakkola et al. 2005).

Although most epidemiologic studies on this topic, including those cited by the IOM (2004), use surrogate measures of mold exposure, such as home dampness, mold odor, or reports of visible mold growth, the health hazard posed by mold is optimally assessed through a description both of the quantity of mold spores and of the different genera (and species when possible) of mold present. Spore concentrations in indoor problem areas may be compared to outdoor levels. In the United States, nationwide and region-specific outdoor benchmarks have been established by the National Allergy Bureau (NAB) Aeroallergen Network of the American Academy of Allergy, Asthma, and Immunology based on historical data (NAB 2006a).

## Endotoxin

Endotoxin refers to soluble lipopolysaccharide fragments that form part of the cell wall of gram-negative bacteria. Inhaled endotoxin causes an inflammatory reaction in humans, especially at high doses, including fever, flulike symptoms, cough, headache, and respiratory distress (Douwes et al. 2003). Chronic exposure to endotoxin at concentrations found in the air in some workplaces is associated with increased risk of upper respiratory infections, airway inflammation, asthma attacks, chronic bronchitis, and hypersensitivity pneumonitis (Rylander 2002). Short-term exposure to endotoxin in the workplace at concentrations > 45 EU (endotoxin units)/m^3^ has been linked to decreased lung function over a single day (Milton et al. 1996). Endotoxin in indoor air is suspected of playing a role in “sick building syndrome” (Rylander 2004).

The association between endotoxin and childhood asthma is complex. Living in homes with moderately elevated concentrations of endotoxin in house dust has been associated with increased risk of wheezing in infants (Park et al. 2001). However, other studies suggest that exposure to endotoxin may decrease risk of childhood atopy (Perzanowski et al. 2006). Overall, the literature to date suggests that exposure to house dust endotoxin early in life may protect from atopic sensitization and IgE-mediated diseases but remains a risk factor for wheezing in infancy (Eder and von Mutius 2004). The association between endotoxin and asthma may also depend on the dose, timing, and genetics of the host (Liu 2002).

Endotoxin-containing bacteria are normally present at low numbers in the indoor and outdoor environment. Indoor dampness is a known risk factor, resulting in increased concentrations of endotoxin and associated health outcomes (Park et al. 2001). The presence of mold in a home is correlated with increased endotoxin levels (Reed and Milton 2001). Stagnant water and sewage can contain high levels of endotoxin and can be a major source of exposure in outdoor environments (Thorn et al. 2002).

For this study, we assessed the potential respiratory health threats due to mold and endotoxin for workers and returning residents in the New Orleans, Louisiana, area after extensive flooding of the city due to levee breaches after Hurricanes Katrina and Rita.

## Materials and Methods

We conducted air sampling in the city of New Orleans and in the neighboring towns of Metairie, Chalmette, and Mandeville, Louisiana, in October and November 2005. The first sampling event took place 16–19 October 2005 and included mold spores and endotoxin. The second, on 13–16 November 2005, focused only on mold spores.

The mean daily temperature and relative humidity during the 4-day October sampling period was 23°C (73°F; range, 16–29°C) and 52% (range, 16–27%), respectively. No precipitation was recorded. During the 4-day November sampling event the mean daily temperature and relative humidity was 21°C (70°F; range, 16–27°C) and 70% (range, 51–89%), respectively. On 15 November, 101 mm (4 in.) of rain was recorded at the New Orleans airport. Outdoor mold sampling data during the rainfall were unusable, and extrapolations were based on the remaining data.

### Monitoring sites

**Figure 1:**
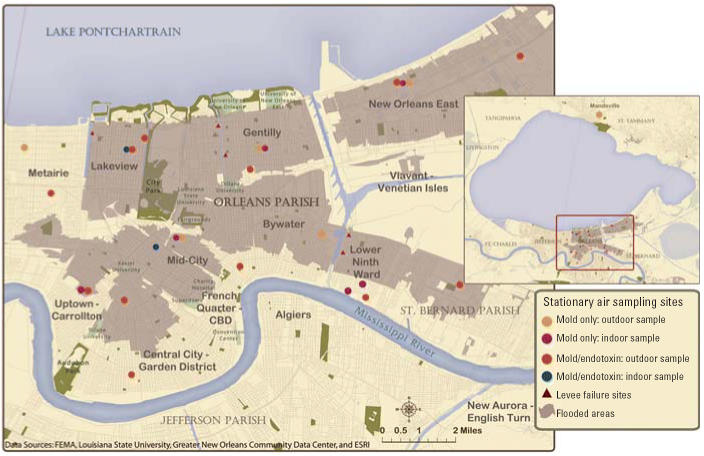
Mold and endotoxin sampling sites, New Orleans area. Data from Environmental Systems Research Institute (ESRI 2005), Federal Emergency Management Agency (FEMA 2006), Greater New Orleans Community Data Center (2002), and Louisiana State University (2005).

We established 23 stationary outdoor monitoring sites for bio-aerosols in residential neighborhoods of the Greater New Orleans area. Outdoor sites represented a variety of conditions and included two nonflooded comparison sites within the city, and three sites more distant from the flooding in the nearby towns of Metairie and Mandeville (Figure 1). Eight indoor sites represented homes that had undergone varying degrees of flooding and remediation. The property owners of each site provided written or verbal permission to enter their property.

Each outdoor site was categorized as “flooded,” “not flooded,” or “distant from flooding.” Indoor sites were categorized according to the degree of flooding and the level of remediation. Two indoor sites were “minimally flooded,” with < 4 cm of water in the living space; one of these was inhabited. The remaining six indoor sites were “severely flooded” with a history of water more than 1 m deep in the living space; none were currently inhabited. The severely flooded indoor sites were further categorized as “unremediated,” “partially remediated,” or “fully remediated.” Unremediated homes contained all contents and were undisturbed since the flooding. Partially remediated homes had furniture and carpets removed and some removal of mold on the walls, such as removal of some drywall or visible evidence of scrubbing. The fully remediated homes had all furniture, carpets, and interior walls removed down to the studs; in some cases the flooring and studs had been sanded and mildewcide may have been applied. Most homes had some windows or doors open, either because they were broken or in an effort to ventilate the interior; the sampling teams caused minimal disturbance of the interior to minimize reaerosolization. When possible, sampling equipment was placed near the center of the first floor of the home, away from open windows or doors.

### Mold sampling

The sampling equipment was placed in wind-protected locations (both outdoor and indoor) on a flat surface away from walls and other obstacles. The orifices of the spore traps were at a height of 0.5–1 m above the floor or ground level. Hirsttype volumetric slit impactors (continuous recording air sampler for glass slides, model 9100; Burkard Manufacturing Co. Ltd., Rickmansworth, UK) measured indoor and outdoor spore concentrations (Hirst 1952). The spore traps had 2-mm × 14-mm slit inlets and operated at an airflow rate of 10 L/min. Particles accumulated on a 48-mm length of transparent, adhesive-coated tape (Melinex, 200 gauge; Burkard Manufacturing Co. Ltd.) that was fixed onto a glass slide with glycerol jelly. The advancement of the glass slide was adjusted according the sampling time either to 6 or 24 hr. Silicon grease (no. 280A; Dow Corning, Midland MI), diluted with xylene, was used as adhesive on the impaction surface. The slide movement was adjusted to 8 mm/hr for 6 hr and to 2 mm/hr for 24-hr sampling time. In flooded areas where no electrical power was available, we used deep-cycle marine batteries connected to inverters to power the sampling equipment.

One investigator (M.H.-K.) examined the samples by reading the particle traces transversely at 2- to 4-mm intervals in a compound microscope with a magnification of 1,000× For each transverse trace, spores within 20 evenly spaced, 92-μm^2^ fields were counted and identified to the extent feasible microscopically (Käpylä and Penttinen 1981). Spore concentrations were available for each 30- or 60-min increment to examine temporal variations of total spore concentrations, and of individual taxa during the sampling periods. The results were extrapolated to 24-hr estimated mold spore concentrations expressed as spores per cubic meter of air. Time discrimination was 1 hr for the 24-hr samples and 15 min for the 6-hr samples (Sterling et al. 1999).

### Endotoxin sampling

We collected samples for endotoxin analysis on 37-mm Teflon filters with 2-μm pore size (model 7592-104; Whatman Inc., Clifton, NJ) at a flow-rate of 10 L/min using a Leland Legacy pump (SKC Inc., Eighty Four, PA) for 6 hr during daytime hours. Sampling pumps were calibrated before and after each sampling event, and the batteries were recharged each night. The average flow rate was multiplied by the sampling time to generate a total volume for each sample. The sampling equipment was placed in wind-protected locations (both outdoor and indoor) on a flat surface away from walls and other obstacles. The orifices of the cartridges were at a height of 0.5–1 m above floor or ground level.

We used the kinetic *Limulus* assay, as described by Milton et al. (1992) and Park et al. (2000), to determine the presence of endotoxin in airborne samples. The kinetic *Limulus* assay method is an *in vitro* biological assay in which *Limulus* amebocyte lysate (LAL) is activated in the presence of endotoxin. In this chromogenic reaction, the LAL enzymes cause the release of a chromophore that is detected by a spectrophotometer. LAL was purchased from BioWhittaker (Walkersville, MD), control standard endotoxin from Associate of Cape Cod (Woods Hole, MA), and reference standard endotoxin from the U.S. Pharmacopeia Inc. (Rockville, MD). All glassware was baked at 270°C at least for 30 min before use. The filter and dust samples were extracted by sonication in 5 mL of tri-ethylamine phosphate buffer, pH 7.5, for 1 hr. After extraction, the sample was serially diluted in endotoxin-free test tubes and placed in a 96-well polystyrene microplate (Associate of Cape Cod). Control standards and blanks were also loaded on the microplate for each assay. LAL was then added to each well, and the plate was monitored every 30 sec for a period of 120 min. The absorbance wavelength was 405 nm, and the incubation temperature was 37°C. The concentration of endotoxin is proportional to the magnitude of the reaction rate and the color change. The standard and sample dilution curves are compared using an estimated parallel-line bioassay analysis to determine the validity of the assay. Results are reported in endotoxin units.

We performed standard quality control measures. Two sets of standard endotoxin solutions and one set of reagent blanks were run on each endotoxin plate, which also contained 13 samples. Both laboratory and field blanks have been collected and analyzed by these methods in our laboratory over the past 6 years. None of > 260 laboratory blank filters has had detectable levels of endotoxin (i.e., all were < 0.0001 EU, which would be < 0.00004 EU/m^3^) in 6 hr of sampling, so no pretreatment of the filters was deemed necessary.

## Results

### Mold

Mold spore concentrations in all of the samples were high to very high according to NAB national benchmarks (NAB 2006a), ranging from approximately 21,000 to 102,000 spores/m^3^ of air outdoors, and from 11,000 to 645,000 spores/m^3^ indoors. Although the mold sampling was conducted during two visits and the sampling time was longer during the second visit, the mean outdoor spore concentrations in the flooded areas between the October sampling (59,706) and the November sampling (80,703) were not statistically different (*p* = 0.14). Therefore, the results of both sampling trips are pooled.

Sampling was conducted at some sites for approximately 6 hr during the daytime, whereas others were collected for up to 24 hr. For those sites where the sampling duration exceeded 6 hr, we also assessed a 6-hr daytime concentration representing the time period from 0900 to 1500 hr (Table 1). Although this daytime spore concentration differed from the 24-hr concentration at each site, the difference was not consistent in either magnitude or direction, and the mean difference was not significantly different from zero (*p* = 0.8). Therefore, 24-hr spore concentrations (measured or estimated based on shorter sampling periods) were used as the unit for all comparisons.

During the October sampling, in addition to the 6-hr average, we assessed spore concentrations for 30-min intervals. Maximal 30-min spore concentrations ranged from 26 to 251% higher than the mean for the entire sampling period. The highest 30-min concentrations were 1,002,456 spores/m^3^ indoors and 259,200 spores/m^3^ outdoors.

**Figure 2:**
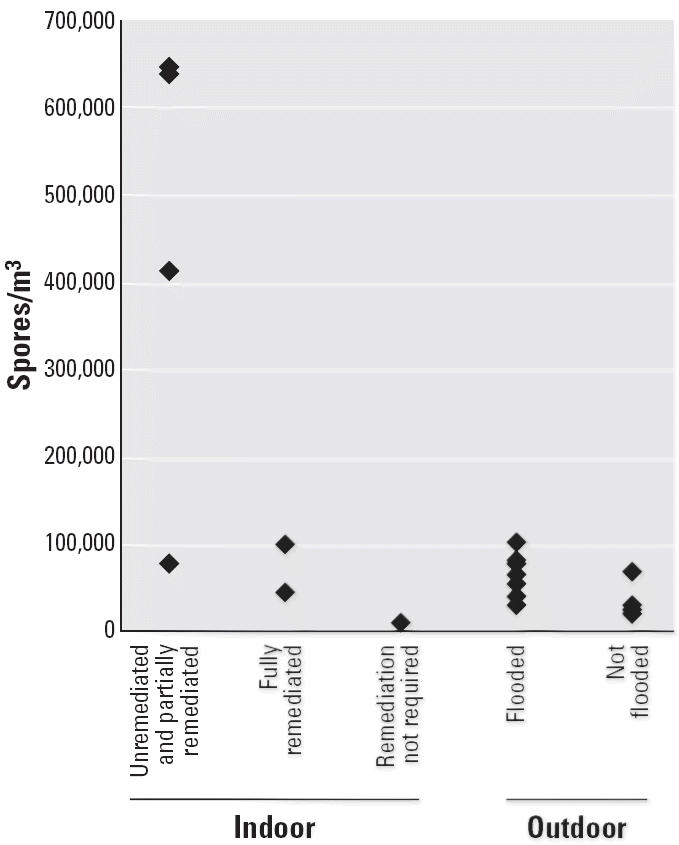
Sampling sites and estimated daily mold spore concentration.

Mold spore concentrations differed significantly by location. The mean outdoor spore concentration in flooded areas was approximately twice the concentration in nonflooded areas (66,167 vs. 33,179 spores/m^3^; *p* < 0.05) (Figure 2). A mean background concentration for the region of 23,835 spores/m^3^ [95% confidence interval (CI), 17,664–30,006] was estimated from contemporaneous sampling in nonflooded areas of nearby towns.

The ambient spore concentrations measured in nonflooded sections of New Orleans that were adjacent to flooded neighborhoods were also high according to NAB national benchmarks (NAB 2006a). The spore concentrations measured at two sites located on nonflooded streets within the city were greater than the concentrations found in nonflooded areas outside the city but were generally less than the spore concentrations measured in the flooded areas (Table 1).

In the flooded areas, the mean concentration of mold spores inside homes (320,005 spores/m^3^) was significantly higher than the mean concentration outdoors (66,167 spores/m^3^; one-tailed *p* < 0.05). However, the two homes that were minimally flooded had spore concentrations that were lower than outdoor samples taken at the same locations (11,192 vs. 81,432 and 10,881 vs. 81,788 spores/m^3^).

We assessed differences in spore concentrations between severely flooded homes that had undergone different levels of remediation at the time of sampling (Table 2). The highest concentration (644,760 spores/m^3^) was measured inside an unremediated home, and the lowest among the homes that were severely flooded (45,050 spores/m^3^) was in a fully remediated home. The mold concentration in the latter home was lower than the concentration outdoors at the same location (62,971 spores/m^3^).

A total of 45 different fungal taxa were identifiable among the samples. The most common genera of mold detected in both indoor and outdoor samples were *Cladosporium* and *Aspergillus*/*Penicillium*. We were unable to confidently differentiate the *Aspergillus* spores from *Penicillium* spores because of their morphologic similarity. We were also unable to determine the specific species present within these genera. Other fungi that significantly contributed to the total spore count at one or more locations include, *Alternaria*, Ascomycetes, *Aureobasidium*, Basidiomycetes, *Chaetomium*, *Curvularia*, *Ganoderma*, smuts, *Stachybotrys*, *Ustilago*, *Wallemia*, and yeasts (Table 3).

Outdoors in the flooded areas, *Cladosporium* constituted about 20–50% of spores found, and *Aspergillus*/*Penicillium* comprised about 20–70% of spores at any given location. In combination, these genera accounted for between 60 and 90% of all spores in outdoor samples. At outdoor nonflooded sites, the dominant genus was *Cladosporium* (70–75%); *Aspergillus*/*Penicillium* was far less common (Table 1). The mean number of identifiable fungal groups in individual outdoor samples was 19 (range, 14–26) in flooded areas and 17 (range, 11–22) in nonflooded areas.

Indoors in flooded homes, the dominant mold was *Aspergillus*/*Penicillium*, accounting for > 70% of fungal spores identified. The minimally flooded homes tended to have fewer spore types represented in the samples (8–11 taxa) compared with the severely flooded homes irrespective of remediation status, which had 16–26 different taxa per home. The fully remediated homes had spore taxa distributions similar to those in outdoor air, except that in four flooded homes (including one fully remediated home) we detected airborne spores of *Stachybotrys* species (Table 2). In one flooded home, the 30-min maxima revealed a *Stachybotrys* spore concentration of up to 324,648 spores/m^3^ during daytime hours. Because of the elevated levels of all mold taxa at these sites, the relative percentage of *Stachybotrys* spores was small.

### Endotoxin

The endotoxin levels in the greater New Orleans area ranged from 0.6 to 8.3 EU/m^3^ (Table 4). There were no significant differences between mean concentrations measured at sites in flooded and nonflooded areas or between indoor and outdoor sites. The mean outdoor endotoxin concentration in flooded areas was 3.9 EU/m^3^ (95% CI, 2.2–5.6), whereas the mean in nonflooded areas was 4.2 EU/m^3^ (95% CI, 1.5–6.9). Endotoxin levels in the two indoor samples from flooded homes were 4.5 and 7.3 EU/m^3^, which was in the same range as the outdoor concentrations. Linear regression found no evidence of an association between the concentration of endotoxin and of mold measured at the same sites (*r*^2^ = 0.097; *p* = 0.32).

## Discussion

### Mold

The NAB is the primary source for nationwide data on outdoor fungal spore concentrations. This network monitors ambient spore concentrations throughout the United States from rooftop height using mainly Hirsttype 7-day recording volumetric spore traps, which provide results directly comparable to those in this study. The NAB defines spore concentrations as “low,” “moderate,” “high,” or “very high” compared to the 50th, 75th, and 99th percentiles of historical ambient mold spore concentrations in the network (NAB 2006a). Ambient spore concentrations > 50,000 spores/m^3^ are defined as “very high” (Table 5) and exceed the 99th percentile nationwide. Ten of the 13 samples (77%) taken within the flooded areas of New Orleans exceeded 50,000 spores/m^3^. In addition, because the dominant spore types found in this study were different from the normal mix of fungi in ambient air, on which the NAB bases its guidelines, it is possible that the health effects for those with allergies or asthma may be greater or less than would be predicted based on the spore concentrations alone.

The only prior published data on mold spore concentrations in New Orleans dates to 1968 and used a methodology not comparable with the present study (Salvaggio and Seabury 1971). Contemporaneous outdoor mold spore concentrations are available from other Gulf Coast locations. The NAB sampling station in Baton Rouge, Louisiana, 112 km (70 miles) from New Orleans, reported spore concentrations of 16,111–23,433 spores/m^3^ in October 2005, which is similar to the range we obtained in nonflooded sampling sites just outside New Orleans. Other NAB sampling stations in the southeastern and south-central United States reported mean spore concentrations during the October–November 2005 time period of 6,696 spores/m^3^ in Tampa, Florida; 1,300 spores/m^3^ in Houston, Texas; and 15,016 spores/m^3^ in Fort Smith, Arkansas (NAB 2006b).

In the indoor environment, there is no consensus methodology for monitoring airborne spore concentrations. Results have been reported in colony-forming units (CFUs) and in spore concentrations; there are a variety of sampling methodologies and equipment, and sampling times vary widely. These inconsistencies make it difficult to compare results across studies. Although most studies in the published literature use CFUs as the indoor exposure metric, previous investigations have revealed a significant, yet variable, potential for underreporting when only CFUs are counted (Burge et al. 1977). Sampling of total spore concentrations is more comprehensive because nonviable spores can trigger allergic reactions or carry mycotoxins, and results are comparable between indoor and outdoor locations.

Reviews of the published literature have reported that indoor background spore concentrations in residences not affected by mold or moisture average 913 spores/m^3^ and range from 68 to 2,307 spores/m^3^ (Gots et al. 2003). Some investigators have used retrospective data to differentiate between mold-affected and unaffected buildings. One review of data from 625 buildings in Southern California found mold concentrations < 4,000 spores/m^3^ in 90% of buildings with no history of water damage and no visible dampness or mold. In contrast, indoor spore concentrations ranged from 200 to 2 × 10^6^ spores/m^3^ in buildings with water damage and visible mold growth (Baxter et al. 2005). The authors suggested 1,300 spores/m^3^ as the cutoff that would minimize false positives and false negatives in defining a “moldy” residential building. By this criterion, all the homes sampled in New Orleans would be defined as moldy.

Other investigators have monitored air inside homes after flooding. One study in Colorado monitored eight homes that had been completely remediated—defined as replacement of wet carpets, dry wall, and sub-floors; washing of nonstructural surfaces with bleach; and air-drying (Fabian et al. 2005). Although the investigators used a different methodology than that used in the present study, sampling 2–3 months after cleaning and reoccupation revealed that seven of the eight homes still had significantly higher concentrations of total airborne microorganisms, and six homes had higher levels of culturable fungi, compared with concentrations in outdoor air at the same location. Thus, there is some concern that the elevated airborne fungal concentrations measured in the present study may persist even after remediation.

There are unresolved uncertainties regarding airborne mold spore concentrations in previously flooded areas of Greater New Orleans. This study took two snapshots in time, approximately 6 weeks after Hurricane Katrina, and again after approximately 10 weeks. The amount of variability over time and space has not been fully characterized and would be difficult to characterize without a sustained and major sampling effort. It is difficult to know how representative these samples are of the conditions inside homes that were not sampled, or outdoors at other times or locations, and under other meteorologic conditions. The strengths of this study include longer sampling times than in many other studies (6–24 hr samples as opposed to 5–15 min samples). Because fungi (including molds) release their spores at different times during the day, brief sampling times may miss potentially important episodes of spore release and are less accurate for estimating daily spore concentrations.

### Endotoxin

The endotoxin concentrations measured in this study were slightly higher than those that have been reported using the same method in residential environments and outdoor air in other parts of the country. A California study reported an ambient average concentration of 0.4 EU/m^3^, with a peak level of 5.4 EU/m^3^ (Mueller-Anneling et al. 2004). No ambient background endotoxin concentrations are available for the New Orleans area before the flooding. In indoor air, a study of 15 homes in Boston, Massachusetts, reported a mean concentration of 0.64 EU/m^3^ (Park et al. 2000). The Fresno (California) Asthmatic Children’s Environment Study reported a slightly higher mean concentration of 1.89 EU/m^3^ in homes without pets and of 2.70 EU/m^3^ in homes with pets (Hammond et al. 2003). These comparisons should be viewed with some caution, however, because prior investigations have reported significant problems with comparing endotoxin concentrations across studies, especially if the studies are done in different laboratories (Reynolds et al. 2005).

There are no regulatory standards for endotoxin, although some thresholds have been proposed. The Dutch Expert Committee on Occupational Standards (1998) recommended a limit value for workers of 50 EU/m^3^ over 8 hr. Mueller-Anneling et al. (2004) extrapolated a threshold ambient 24-hr concentration for acute airway obstruction of 17 EU/m^3^. In contrast, the American Conference of Governmental Industrial Hygienists (ACGIH) has set a relative limit value (RLV) whereby endotoxin concentrations > 10 times background are considered a concern in an environment where there are complaints of respiratory symptoms, and concentrations 30 times background should be avoided at all times (Macher 1999). Our sampling failed to reveal any areas where airborne endotoxin concentrations approached these threshold numbers during the time period when the sampling was done. Although the endotoxin concentrations ranged > 10-fold in this study, there were no systematic differences between indoor and outdoor concentrations, or between concentrations in flooded and non-flooded areas that would fit the criteria described in the ACGIH RLV (Macher 1999).

The lack of association between flooding and endotoxin concentrations, as well as between endotoxin and mold concentrations, was surprising. Endotoxin does not become airborne as readily as mold spores. Therefore, the lack of elevated air concentrations may reflect the study protocol, which specified minimum disturbance of the area during sampling. A CDC study of 20 New Orleans homes in late October 2005 found mean endotoxin concentrations of 23.3 EU/m^3^ indoors and 10.5 EU/m^3^ outdoors (CDC 2006). These concentrations were higher than those found in our study and were consistent with the hypothesis that gram-negative bacterial growth was occurring inside the flooded homes. It remains likely that people doing remediation work in or around flooded homes could cause release of endotoxin into the air, resulting in elevated exposures.

## Conclusion

The high concentrations of mold measured indoors and outdoors in the New Orleans area are likely to be a significant respiratory hazard that will be important to monitor over time. This study represents an early assessment in the aftermath of an important environmental disaster, which may offer a point of comparison for future work. In the meantime, these results indicate a need to undertake widespread public education efforts to encourage use of appropriate personal protective equipment and exposure mitigation techniques among workers and returning residents in order to prevent respiratory morbidity. Survey results suggest that nearly half of returning residents and one-third of remediation workers are not consistently wearing personal protective equipment (CDC 2006). The mold concentrations detected in this study also raise potentially significant concerns for long-term health effects in the pediatric population that may be returning to the flooded areas.

## Figures and Tables

**Table 1:** Daily mean fungal spore concentrations and predominant taxa: outdoor sampling sites.

		Spore concentration (spores/m^3^)		Spore taxa (%)	
Sample	Flooding extent	Estimated full day (24 hr)	Measured daytime (6 hr)	*Cladosporium*	*Aspergillus/Penicillium*
1–7	Distant from flooding	30,132	30,132*^a^*	41	41
2–1	Distant from flooding	20,718	28,026	69	8
2–8	Distant from flooding	20,655	22,302	76	4
1–4	Not flooded	68,040	68,040	46	25
1–11	Not flooded	26,352	26,352	46	33
1–13	Flooded	102,222	102,222	22	71
2–11	Flooded	100,813	100,813	52	29
2–6	Flooded	81,788	66,204	61	27
1–3	Flooded	81,432	81,432	18	61
2–10	Flooded	77,241	110,592	56	23
1–6	Flooded	76,626	76,626	54	16
1–5	Flooded	66,582	66,582	36	40
2–3	Flooded	62,971	62,971	76	16
1–1	Flooded	54,756	54,756	43	20
1–2	Flooded	53,406	53,406	51	19
1–9	Flooded	40,068	40,068	29	57
1–8	Flooded	31,806	31,806	48	31
1–14	Flooded	30,456	30,456	53	29

a Spore concentration estimated from 5-hr sample.

**Table 2:** Daily mean fungal spore concentrations and predominant taxa: indoor sampling sites.

			Spore concentration (spores/m^3^)		Spore taxa (%)		
Sample	Flooding extent	Remediation level	Estimated full day (24 hr)	Measured daytime (6 hr)	*Cladosporium*	*Aspergillus/Penicillium*	*Stachybotrys*
2–4	Minimal flooding	Not applicable	11,192	12,150	59	31	ND
2–5	Minimal flooding	Not applicable	10,881	12,744	39	50	ND
2–2	Flooded	Full remediation	45,050	74,952	38	48	ND
2–9	Flooded	Full remediation	99,792	99,792	39	39	< 1
2–12	Flooded	Partial remediation	78,521	31,320	24	67	< 1
2–7	Flooded	Partial remediation	413,870	416,178	26	65	< 1
1–10	Flooded	Partial remediation	638,037	638,037	6	83	ND
1–12	Flooded	Unremediated	644,760	644,760*^a^*	7	82	2

ND, not detected.

a Spore concentration estimated from 4-hr sample.

**Table 3:** Fungal taxa identified at > 1% of total spore count.

	Outdoor		Indoor	
Mold taxa	Flooded	Nonflooded	Flooded	Minimally flooded
*Alternaria* species	X	X		
Ascomycetes	X	X	X	X
*Aspergillus*/*Penicillium* species	X	X	X	X
*Aureobasidium* species	X		X	X
Basidiomycetes	X	X		
*Chaetomium* species			X	
*Cladosporium* species	X	X	X	X
*Curvularia* species	X		X	
*Ganoderma* species	X			
Smuts	X	X	X	
*Stachybotrys* species			X	
*Ustilago* species	X	X		
*Wallemia* species			X	
Yeast			X	

Although the remaining 31 taxa each constituted < 1% of the total spore count at any individual site, the concentrations of many were substantial given the overall high levels of spores found.

**Table 4:** Endotoxin concentrations: indoor and outdoor sampling sites.

		Sampling	Endotoxin
Sample	Flooding extent	location	(EU/m^3^)
1–7	Distant from flooding	Outdoor	5.0
1–4	Not flooded	Outdoor	6.1
1–11	Not flooded	Outdoor	1.5
1–1	Flooded	Outdoor	8.3
1–2	Flooded	Outdoor	0.6
1–3	Flooded	Outdoor	5.7
1–5	Flooded	Outdoor	1.8
1–6	Flooded	Outdoor	1.8
1–8	Flooded	Outdoor	6.2
1–9	Flooded	Outdoor	3.0
1–13	Flooded	Outdoor	5.4
1–14	Flooded	Outdoor	2.0
1–10	Flooded	Indoor	4.5
1–12	Flooded	Indoor	7.3

**Table 5:** NAB Aeroallergen Network outdoor mold spore concentration classifications.

Concentration (spores/m^3^)*^a^*	NAB classification
0	Absent
1–6,499	Low
6,500–12,999	Moderate
13,000–49,999	High
> 50,000	Very high

Data from NAB (2006a).

a Daily mean spore concentrations per cubic meter.
